# Utilisation of ISA Reverse Genetics and Large-Scale Random Codon Re-Encoding to Produce Attenuated Strains of Tick-Borne Encephalitis Virus within Days

**DOI:** 10.1371/journal.pone.0159564

**Published:** 2016-08-22

**Authors:** Lauriane de Fabritus, Antoine Nougairède, Fabien Aubry, Ernest A. Gould, Xavier de Lamballerie

**Affiliations:** 1 UMR "Emergence des Pathologies Virales" (EPV: Aix-Marseille University—IRD 190—Inserm 1207—EHESP), Marseille, France; 2 Institut Hospitalo-Universitaire Méditerranée Infection, Marseille, France; International Centre for Genetic Engineering and Biotechnology, ITALY

## Abstract

Large-scale codon re-encoding is a new method of attenuating RNA viruses. However, the use of infectious clones to generate attenuated viruses has inherent technical problems. We previously developed a bacterium-free reverse genetics protocol, designated ISA, and now combined it with large-scale random codon-re-encoding method to produce attenuated tick-borne encephalitis virus (TBEV), a pathogenic flavivirus which causes febrile illness and encephalitis in humans. We produced wild-type (WT) and two re-encoded TBEVs, containing 273 or 273+284 synonymous mutations in the NS5 and NS5+NS3 coding regions respectively. Both re-encoded viruses were attenuated when compared with WT virus using a laboratory mouse model and the relative level of attenuation increased with the degree of re-encoding. Moreover, all infected animals produced neutralizing antibodies. This novel, rapid and efficient approach to engineering attenuated viruses could potentially expedite the development of safe and effective new-generation live attenuated vaccines.

## Introduction

Many emerging infectious diseases are caused by arthropod-borne viruses (arboviruses) of which pathogenic flaviviruses, such as yellow fever virus (YFV), dengue virus, Japanese encephalitis virus (JEV), West Nile virus and tick-borne encephalitis virus (TBEV) are major human pathogens [1–3]. As history has shown, vaccination is a powerful method to combat viral diseases [4–7]. Live attenuated vaccines can provide effective and affordable protection against flaviviral infections. For example, one dose of the widely used YFV or JEV vaccines provides long-lasting immunity. Live attenuated virus strains have been obtained in the past using empirical methods such as serial sub-culture of wild-type (WT) viruses [8–10]. In view of the growing public demand in terms of protection against emerging diseases and also concerns for drug safety, effective, safe and rapid approaches are needed to produce new-generation live attenuated vaccines.

The large-scale codon re-encoding procedure is a recently developed attenuating technique that modifies the nucleic acid composition of large coding regions without modifying the encoded proteins, by introducing a large number of slightly deleterious synonymous mutations. This method of attenuation takes generic advantage of live attenuated vaccines but also enables precise modulation of the attenuated phenotype. It also alleviates the generation of undesirable new biological properties because re-encoded viral genomes encode identical proteins [11]. This procedure has already been successfully applied to a wide range of RNA viruses [12–22].

We previously applied random large-scale codon re-encoding to the TBEV Oshima 5–10 strain, a highly neurovirulent strain for mice which belongs to the Far Eastern TBEV subtype [23–25]. Re-encoded virus was derived from the WT virus by substituting a cassette of approximately 1.4kb located in the NS5 coding region which contained 273 randomly introduced synonymous mutations. This re-encoded TBEV strain displayed an attenuated phenotype when tested in a laboratory mouse model and induced protective immunity in mice subsequently challenged with WT virus [22].

To date, all studies of codon re-encoding have used infectious clones to generate WT and attenuated viruses [12–22]. However, construction of such infectious cDNA clones is often time-consuming, laborious and unpredictable. Recently, a new bacterium-free approach, called ISA (Infectious subgenomic amplicons), was described to generate infectious single-stranded positive-sense RNA viruses [26]. This method avoids the need for cDNA cloning procedures and shortens the time to produce engineered virus. In the present study, we demonstrate the feasibility of producing attenuated viruses using the ISA method combined with random codon re-encoding.

## Materials and Methods

### Cell lines and animals

Baby hamster kidney (BHK21) cell line (ATCC, number CCL10) and mouse (L929) cell line (ATCC, number CCL1) were maintained in Minimum Essential Medium with 7% foetal calf serum (Life Technologies) and 1% Penicillin/Streptomycin (5000U/mL and 5000μg/mL; Life Technologies) at 37°C with 5% CO_2_. Five-week-old C57Bl/6J mice females were purchased from Charles River laboratories.

### Ethics statement

Animal protocols were approved by the ethics committee “Comité d’éthique en expérimentation animale de Marseille—C2EA—14” (protocol number 2504). All *in vivo* experiments were performed in accordance with the European legislation covering the use of animals for scientific purposes (Directive 210/63/EU) and French national guidelines.

### Animal handling

Mice were maintained on a 12h:12h light:dark cycle had ad libitum access to rodent chow and water. Weight and general health of each animal was monitored daily, and all efforts were made to minimize suffering. Acetaminophen was systematically administered in the drinking water (2mg/ml) and buprenorphine (1mg/kg/day, intraperitoneal route) was administered during 24h-48h before euthanasia (cervical dislocation) when animal had one or combination of the following symptoms: hemiplegia, tetraplegia and important weight loss (≥20%). During all experiments, two mice were euthanized at day 15 post-inoculation (one inoculated with the NS5_ISA virus and one with the WT_ISA virus).

### *In silico* re-encoding method

Cassettes of 1,400 and 1,412 bp located in the NS3 and NS5 coding regions respectively were randomly re-encoded as previously described for NS5_IC TBEV strain [22] (Note B in **S1 Text**). Briefly, a computer programme was run to randomly attribute nucleotide codons based on their corresponding amino acid sequence: for example, the amino acid glycine was randomly replaced by GGT, GGC, GGA or GGG. The number and the position of rare codons in primate genomes [27] (*i*.*e*. CGU, CGC, CGA, CGG, UCG, CCG, GCG, ACG), and unique restriction sites were conserved.

### Production of WT and re-encoded TBEVs using the ISA method

This procedure was described in detail by Aubry *et al*. [26]. Briefly, for all produced viruses, complete genomes flanked respectively at the 5' and 3' extremities by the human cytomegalovirus promoter (pCMV) and the hepatitis delta ribozyme followed by the simian virus 40 polyadenylation signal (HDR/SV40pA) were amplified by PCR in three overlapping DNA fragments. The PCR protocol as well as primer sequences were the same as those previously described [26]. All DNA fragments were obtained using the TBEV infectious clone pTBEV-32.11 ic (Figure A in **S1 Text**) (GenBank accession number KF623542) as template with the exception of cDNA fragments containing re-encoded cassettes for which *de novo* synthesized DNA (cloned into pUC57; Genscript) were used as templates. An equimolar mixture of the amplified DNA fragments was transfected into BHK21 cells as previously described [26]. Nine days post-transfection, a cytopathic effect was observed for all viruses and clarified (centrifugation) cell supernatants were passaged once in L929 cells. Cell supernatants were then harvested when complete cytopathic effect was observed (3–5 days), clarified by centrifugation, aliquoted, stored at -80°C and used to perform *in vivo* experiments. The integrity of complete viral genomes on cell supernatants was done as previously described [26].

### *In silico* sequence analysis

Complete open reading frames of TBEVs (n = 85) and other tick-borne flaviviruses (TBFVs) (n = 56) were extracted from GenBank (Note A in **S1 Text**). G+C% and effective number of codons (eNC) were calculated using Codon W v1.3 program [28, 29].

### Competition experiments

Competition experiments were performed with the same procedure as previously described [21]. Briefly, wild-type virus was competed with re-encoded viruses: three initial TCID50 ratios (WT_ISA/NS5_ISA or WT_ISA/NS3NS5_ISA virus: 20/80, 50/50, 80/20) were used to infect cells at a multiplicity of infection (moi) of 0.5. Recovered infectious cell supernatant was sequentially passaged 6 times. At each passage, a moi of 1 was used. Viral RNA extracted from clarified culture supernatant was used to perform two specific quantitative real time RT-PCR assays which target the re-encoded NS5 coding region and which specifically detect either wildtype or re-encoded viruses. The amount of viral RNA was assessed for each virus (Wild-type and re-encoded) and the ratio of the two values was calculated.

### *In vivo* experiments

As described in our previous study [22], five-weeks-old C57Bl/6J female mice were intra-peritoneally inoculated with 200μL containing 2.10^6^ TCID50 of virus. A control group of mice was used (they were intra-peritoneally inoculated with 200μL of PBS). The clinical course of the infection was monitored by following (i) the clinical manifestations of the disease (shivering, humpback, dirty eyes, hemi- or tetra-paresia, hemiplegia or tetraplegia) and (ii) the weight of the mice. Weights were normalized using the average weight of mice of control group; the normalized weight was expressed as percentage of initial weight and calculated using the following formula: (% of initial weight: weight/weight at the day of the inoculation or challenge)–(mean of the % of the initial weight for control mice) +100. Brains and blood samples (intracardiac puncture) were collected from sacrificed mice. After blood centrifugation, serum was aliquoted and stored at -80°C.

Nucleic acid extraction using a mix containing 50μL of serum, 50μL of AVL buffer (Qiagen) spiked with 10μL of MS2 bacteriophage (internal control) was performed using the EZ1 Virus Mini Kit on the EZ1 Advanced XL machine (both from Qiagen).

Brains were collected in 1mL of PBS with a tungsten bead and grounded using a MM300 mixer (Retsch) for 3min at 30cycles/s. The brain suspensions were homogenized with NucleoSpin filters (Macherey-Nagel). The filtrate (30 μL) was mixed with 270μL of RLT buffer, 10 μL of MS2 bacteriophage (internal control) and used for nucleic acid extraction using the EZ1 RNA Tissue Mini Kit on the EZ1 Advanced XL machine (both from Qiagen).

### Real-time quantitative RT-PCR assays

Real-time quantitative RT-PCR (qRT-PCR) assays were performed with SuperScript III Platinium One-Step qRT-PCR kit (Life Technologies) on CFX96 Real-Time System/C1000 Touch Thermal Cycler machine (Biorad) as described previously [22]. Primers and probe sequences are detailed in Table B in **S1 Text**. All samples from mice were spiked with MS2 bacteriophage (internal control) before nucleic acid extraction step and a MS2-specific qRT-PCR assay was performed to monitor extraction, reverse transcription, and amplification steps as previously described [30]. The TBEV assay was used to detect viral RNA. The amount of viral RNA was calculated using a synthetic RNA transcript (detection limit of the assay: 10^4^ copies/mL). Results from mouse brains were normalized using amplification (qRT-PCR) of the housekeeping gene HMBS as described previously [31].

### Tissue-culture infectious dose 50 (TCID_50_) assay

For each determination, a 96-well plate culture of BHK21 cells was inoculated with 150μL/well of serial 10-fold dilutions of clarified (centrifugation) cell supernatant: each dilution was repeated 6 times. The plates were incubated 7 days and read for absence or presence of cytopathic effect in each well. Determination of the TCID_50_/mL was performed using the method of Reed and Muench [32]. Assuming that the re-encoding could modify significantly the appearance of CPE, we used the TBEV real time RT-PCR assay to confirm presence or absence of infectious virus. This assay was performed once for each virus: CPE positive wells were all positive (threshold cycle < 15) while CPE negative wells were all negative or positive with a threshold cycle >35, the value expected after the dilution of the initial RNA yields.

### ELISA test

Sera were incubated for 30min at 56°C. TBEV-specific immunoglobulin G (IgG) antibodies were detected using the Anti-TBE Virus ELISA (IgG) assay (Euroimmun). Sera were diluted 1:64 and then 1:101 before the first incubation using the Sample Buffer. Goat anti-mouse IgG antibodies (Invitrogen) diluted 1:2000 in BSA 0.7% (KPL) as secondary antibodies were used. Plates were read using the Sunrise reader (Tecan) at a wavelength of 450nm.

### Serum neutralisation assay

Sera were incubated for 30min at 56°C. For each serum, a 96-well plate culture of confluent BHK21 cells was inoculated with 50μL/well of wild-type virus (final calculated multiplicity of infection: 0.001) and 50μL/well of a serial 2-fold dilution (first dilution at 1:40) of serum. Each row included 5 wells of serum dilution, a positive control (virus only) and a negative control with neither virus nor serum. The plates were incubated for 7 days and read for the absence or presence of cytopathic effect in each well. The 50% plaque reduction neutralization titre (PRNT50/mL) was determined using the method of Reed and Muench [32].

### Statistical analysis

Kaplan-Meier survival analysis with Mandel-Cox’s Logrank tests, Fisher's exact tests and Student's t tests were performed using SPSS software package (IBM). *p* values under 0.05 were considered significant.

## Results and Discussion

Using the Oshima 5–10 strain and the ISA protocol, we engineered the WT (WT_ISA virus) and two randomly re-encoded viruses in days (nucleotide codons were randomly attributed based on their corresponding amino acid sequence as detailed in the Materials and Methods section). The first re-encoded virus harbored 273 random mutations in the NS5 coding region (NS5_ISA virus). The second harboured these 273 mutations and 284 additional random synonymous mutations in the NS3 coding region (NS3NS5_ISA) (Figure A in **S1 Text)**. The genetic characteristics of the complete coding regions of all these viruses are detailed in Table A in **S1 Text**.

**Fig 1:**
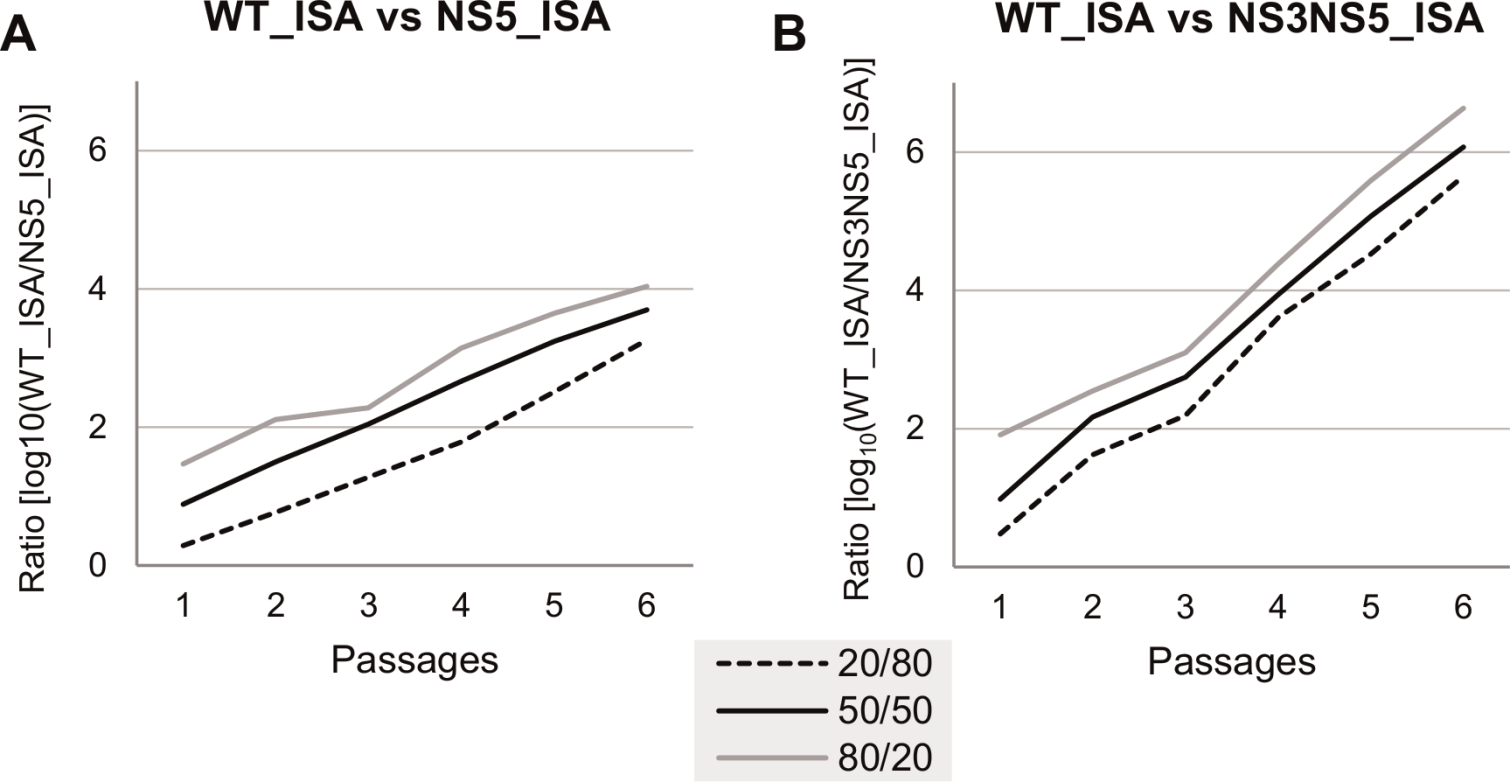
*In cellulo* replicative fitness in BHK21 cells. Results of competition experiments performed using three initial TCID50 ratios (wild-type/re-encoded: 20/80, 50/50, 80/20). Two qRT-PCR assays, each specifically detecting one of the competing virus, enabled to monitor the evolution of the proportion of each virus along 6 passages.

We performed competition experiments to assess the replicative fitness of the engineered viruses since we previously observed that this method provides more sensitive results than growth kinetics [21, 33]. Three initial TCID50 ratios (wild-type/re-encoded: 20/80, 50/50, 80/20) were used to infect BHK21 cells. Infectious cell supernatants were then passaged 6 times in the same cells. Two specific quantitative RT-PCR assays (each specifically detecting one of the competing virus) were used to determine the proportion of each viral genome in cell supernatants at each passage (expressed as log_10_ WT/re-encoded ratio; **Fig 1**). In accordance with our previous work with the Chikungunya virus, we found that the WT_ISA virus was proportionally more fit than re-encoded viruses depending on the degree of re-encoding [21].

We used a previously described mouse model [22, 25, 34, 35]. As previously described, five-weeks-old C57Bl/6J female mice inoculated intraperitoneally with the engineered viruses displayed lower mortality rates when compared with the Oshima 5–10 strain [22, 24]. Therefore, mortality was not used as a comparative criterion.

Three groups of 30 mice were inoculated with 2.10^6^ TCID50 of either WT_ISA, NS5_ISA or NS3NS5_ISA virus. A control group of 14 mice was inoculated with PBS. The viral disease was monitored by following (i) the clinical course of the disease (humpback, shivering, dirty eyes, weak paws, hemiplegia or tetraplegia) and *(ii)* the weight loss (a cut-off of 6% of the initial weight was chosen as a disease recognition criterion, as previously described [22]). Periodically, groups of mice were sacrificed to monitor (i) the viraemia at day 2 post-inoculation (pi), (ii) the neuroinvasiveness at days 7 and 43 pi and (iii) the immune response by performing viral serology at day 43 pi. Viraemia and neuroinvasiveness were assessed by quantifying viral RNA in sera and brain suspension samples using a quantitative real-time RT-PCR assay.

**Fig 2:**
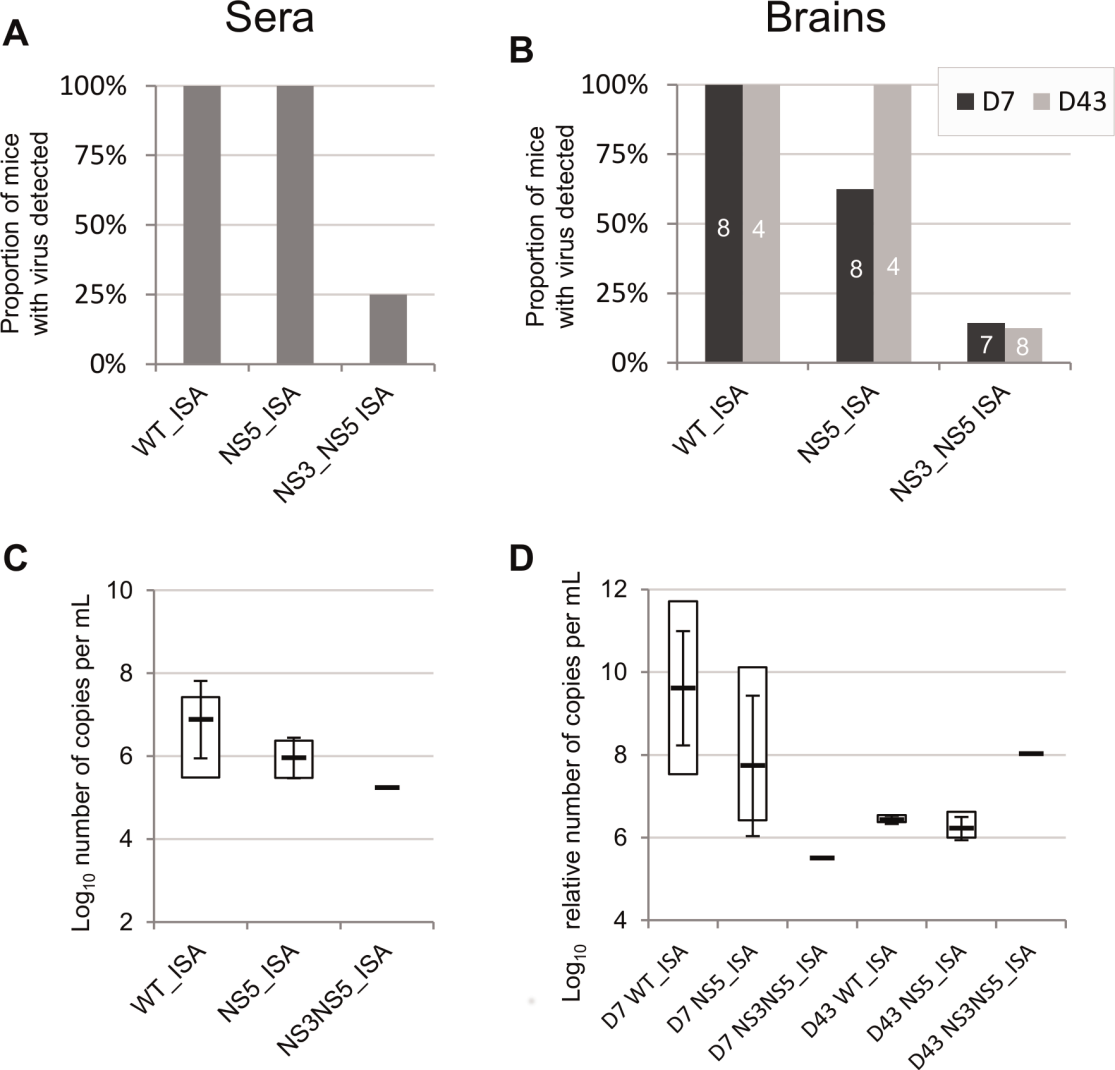
**Proportion of mice with virus detected in the serum (A) or in the brain (B) and viral RNA yields in sera (C) and in the brain (D) by qRT-PCR.** For each group, four sera were sampled at 2 days pi. Brains were samples at 7 and 43 days pi (D7 and D43 in panels **B** and **D**; the number of mice sampled for each time point is indicated onto each bar in panel *B*). Viral RNA yields are expressed as log_10_ number of copies per mL (sera) or log_10_ relative number of copies per mL (brains) (values were normalized using the housekeeping gene HMBS as detailed in the Materials and Methods section). For viral RNA yields, white squares represent minimal and maximal values. Black lines and error bars represent mean values +/- standard deviation.

**Fig 3:**
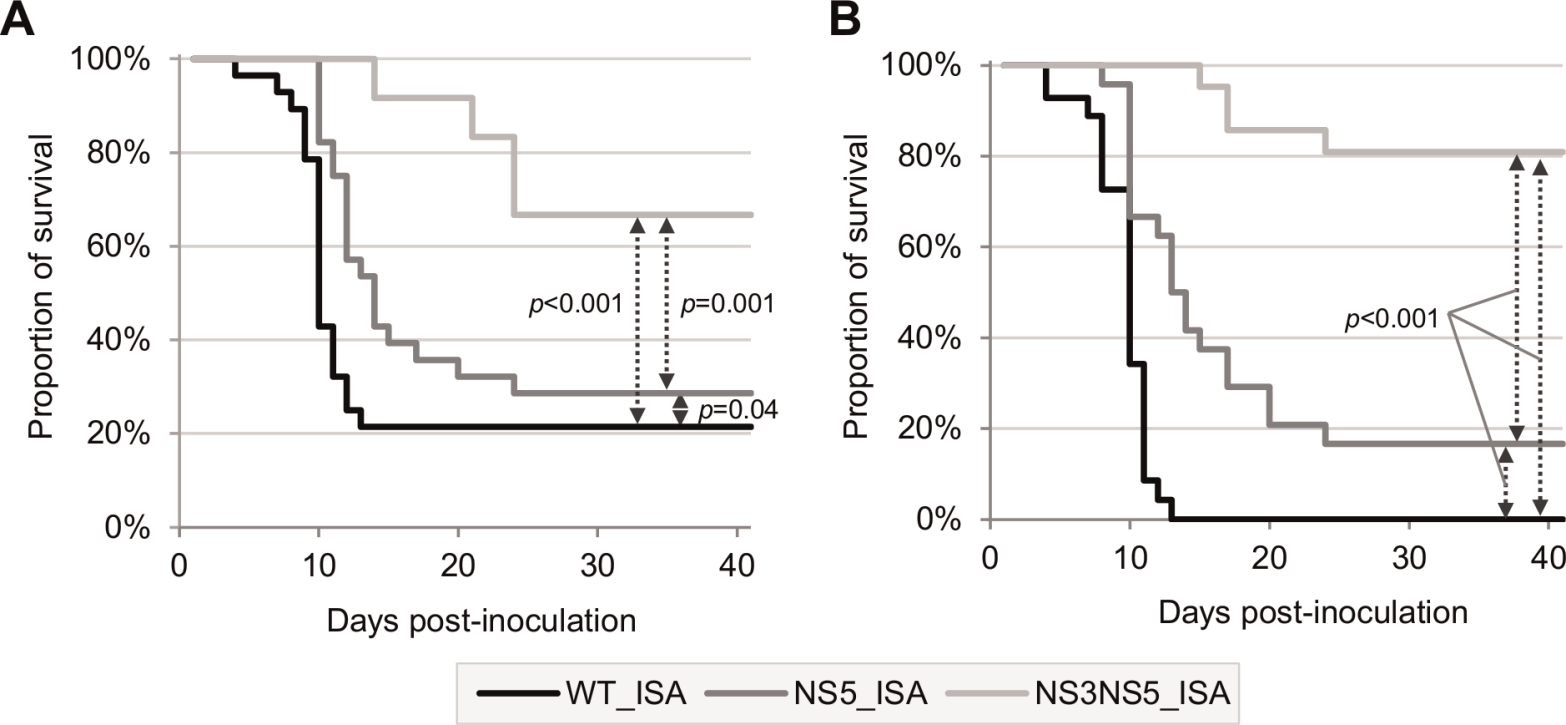
Kaplan-Meier survival analysis using as criteria, a weight loss of more than 6% (A) and appearance of at least one symptom (B).

The proportion of viraemic animals at day 2 pi reached 100% with both WT_ISA and NS5_ISA viruses but decreased to 25% with NS3NS5_ISA virus. However, the difference was not significant (**Fig 2A**). No significant difference was found in term of viral RNA yields detected in sera (**Fig 2C**). The proportion of animals with virus in the brain (neuroinvasion) decreased with the degree of increased re-encoding (**Fig 2B**). The WT_ISA virus produced 100% neuroinvasion at days 7 and 43 pi. The NS5_ISA virus produced 63% and 100% neuroinvasion at days 7 and 43 pi respectively (not significant when compared with the WT_ISA virus). However, NS3NS5_ISA virus displayed significantly lower neuroinvasiveness: 14% and 13% neuroinvasion at days 7 and 43 pi respectively (*p* = 0.001 and *p* = 0.024 when compared with the WT_ISA virus, Fisher exact test). No significant difference was found in term of viral RNA yields detected in brains (**Fig 2D**). The degree of re-encoding correlated with the reduction of the pathogenicity (*i*.*e*. loss of body weight and delayed and less frequent clinical symptoms; see Kaplan-Meier analysis in **Fig 3**).

**Fig 4:**
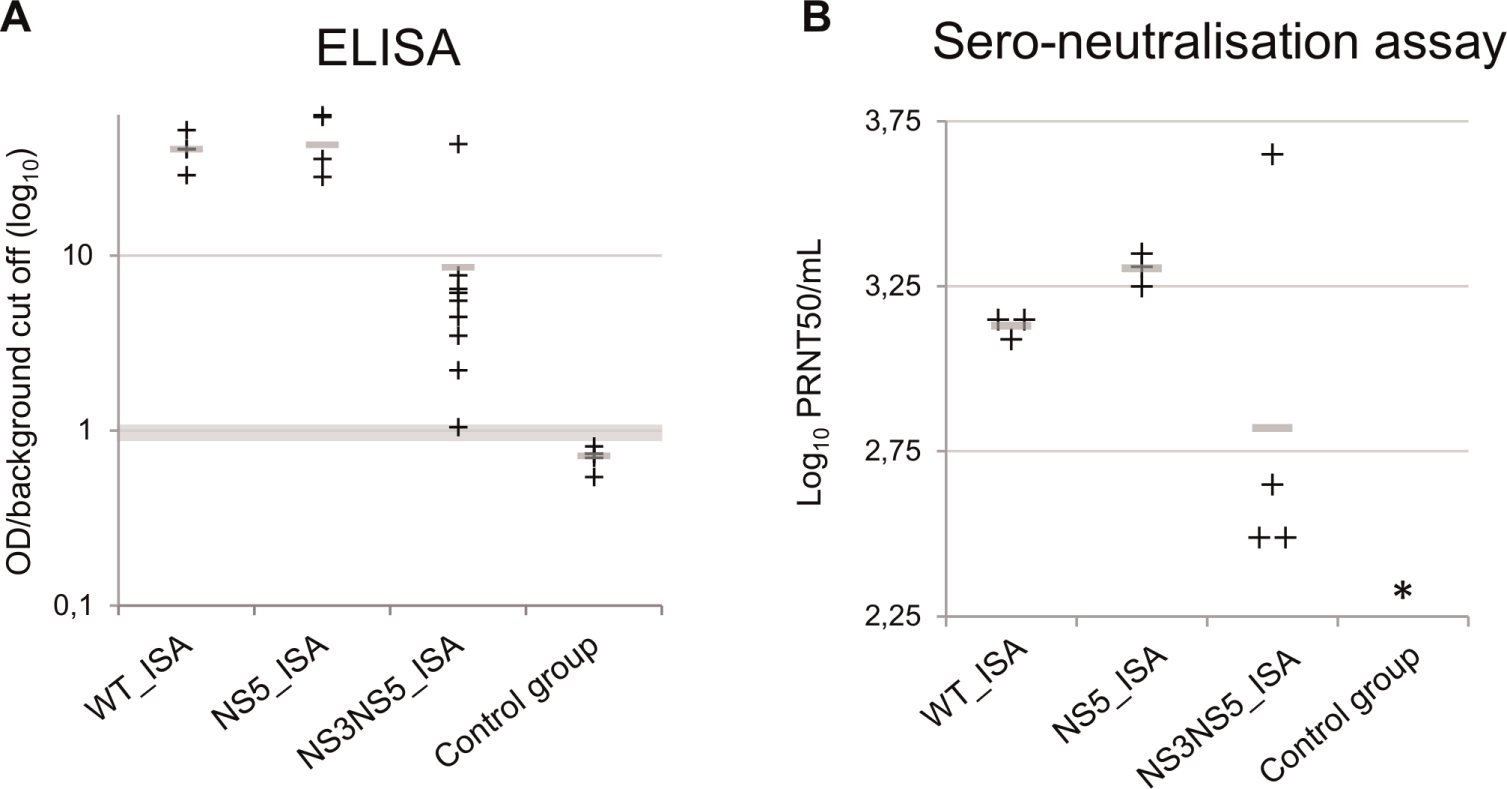
Results of TBEV serology at day 43 post-inoculation. ELISA **(A)** and sero-neutralisation assays **(B)**. Black crosses represent each individual value. Grey bars represent mean values. For ELISA tests, ratios were calculated as follows: OD/background cut off (mean of OD for control group + (3 x SD of OD for control group)). Samples were considered as positive if the ratio was higher than 1.1, negative if under 0.9 and uncertain if between 0.9 and 1.1 (grey zone). * All the sera of the control group tested were negative (*e*.*g*. under the detection threshold of the method (2.25 PRNT/mL)).

Using sera collected 43 days following infection, the presence of TBEV-specific immunoglobulin G (IgG) antibodies and neutralising antibodies was tested using respectively a commercial diagnostic ELISA kit and a serum neutralisation assay. As previously demonstrated, all mice infected by WT_ISA and NS5_ISA viruses produced high levels of neutralising antibodies (**Fig 4**). No neutralising antibodies were detected in control mice. Antibodies were detected in almost all mice infected with NS3NS5_ISA virus: 90% (9/10) when using the ELISA assay and 100% (4/4) when using the neutralisation assay. Overall, levels of antibody were lower than those observed in mice infected by both WT_ISA and NS5_ISA viruses (**Fig 4**). Similar results were found with vaccine strains of measles virus in vivo [36, 37]. Challenge experiments should enable to assess the level of protection against subsequent infection by a wild type virus induced by the NS3NS5_ISA virus.

Our results provide proof of concept for the production of attenuated viruses using the ISA method combined with random codon re-encoding. We used a Far Eastern TBEV strain enabled us to detect the modification of the clinical picture induced by codon re-encoding. However, Far Eastern TBEVs cause more severe infections in humans compared to other subtypes [38]. It would be more opportune to develop a TBEV vaccine candidate using a low-virulence TBEV strain and by adjusting the number of synonymous mutations introduced as well as the administered dose with a view to reducing the encephalitic potential of the virus. In addition, it could be interesting to investigate the phenotypical stability of such attenuated viruses during serial passages in cells and/or mice.

In conclusion, when combined with large-scale random codon re-encoding, this reverse genetic ISA method can rapidly generate attenuated TBEVs. Moreover, since ISA can be applied to other single-strand positive-sense RNA viruses [26] and codon re-encoding has been used to attenuate a variety of RNA viruses [12–22], this combination could accelerate the development of new-generation vaccine candidates. Using the ISA method, wild-type and genetically modified viruses can be produced within days starting from a variety of initial sources including pre-existing infectious clones, *de novo* synthesized DNA genomic sequences or viral RNA. In addition and because each of the three DNA fragments can be exchanged independently, the rapid generation of a multitude of re-encoded viruses using different combinations of re-encoded cDNA fragments offers the possibility to control precisely the attenuation phenotype. Finally, when combined with large-scale codon re-encoding, the ISA method is entirely compatible with other methods of attenuation such as classical site-directed mutagenesis or chimeric approaches.

## Supporting Information

S1 Text Fig A. Schematic representation of the cloning vector pTBEV-32.11 ic (WT_IC) Table A. Genetic characteristics of the complete coding regions of WT_ISA virus, NS5_ISA virus, NS3NS5_ISA virus, 85 tick-borne encephalitis viruses (TBEV) and 56 other tick-borne flaviviruses (TBFV) Table B. Primers and probes used for the real time RT-PCR assays Note A. Sequences retrieved from GenBank Note B. Re-encoded sequences
(PDF)
