# Supplementary material for: Utilisation of ISA Reverse Genetics and Large-Scale Random Codon Re-Encoding to Produce Attenuated Strains of Tick-Borne Encephalitis Virus within Days
Source: PLoS One. 2016 Aug 22;11(8):e0159564. doi: 10.1371/journal.pone.0159564 (PMC4993482; doi:10.1371/journal.pone.0159564)
Supplement: S1 Text — Fig A. Schematic representation of the cloning vector pTBEV-32.11 ic (WT_IC) Table A. Genetic characteristics of the complete coding regions of WT_ISA virus, NS5_ISA virus, NS3NS5_ISA virus, 85 tick-borne encephalitis viruses (TBEV) and 56 other tick-borne flaviviruses (TBFV) Table B. Primers and probes used for the real time RT-PCR assays Note A. Sequences retrieved from GenBank Note B. Re-encoded sequences (PDF) [file pone.0159564.s001.pdf]

Utilisation of ISA reverse genetics and large-scale random codon-re-encoding to produce attenuated strains of tick-borne encephalitis virus within days

Lauriane de Fabritus, Antoine Nougairède, Fabien Aubry, Ernest A Gould and Xavier de Lamballerie

Supporting information

Figure A. Schematic representation of the cloning vector pTBEV-32.11 ic (WT_IC) 1

Table A. Genetic characteristics of the complete coding regions of WT_ISA virus, NS5_ISA virus, NS3NS5_ISA virus, 85 tick-borne encephalitis viruses (TBEV) and 56 other tick-borne flaviviruses (TBFV) 2

Table B. Primers and probes used for the real time RT-PCR assays 2

Note A. Sequences retrieved from GenBank 3

Note B. Re-encoded sequences 4

Figure A. Schematic representation of the cloning vector pTBEV-32.11 ic (WT_IC)

Coding (white rectangles) and non-coding (black rectangles) regions which represent the complete genome of the TBEV WT_IC were flanked in 5’ and 3’ by the pCMV and the HDR/SV40pA, and inserted into a modified pBR322 plasmid. The re-encoded cassettes are represented by a grey rectangle flanked by the restriction sites XmaI and BglII for the NS3 cassette and SacII and SalI for the NS5 cassette.

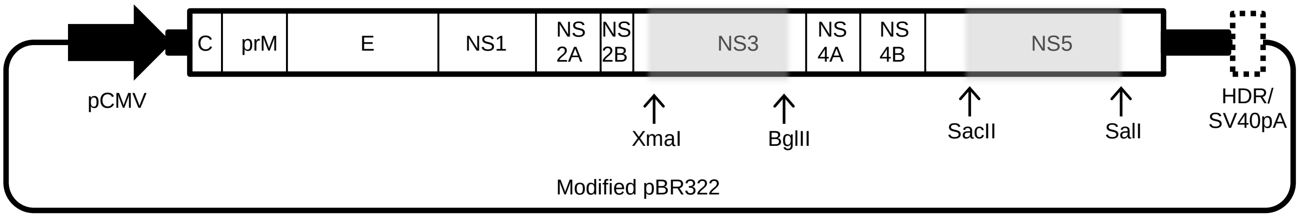

Table A. Genetic characteristics of the complete coding regions of WT_ISA virus, NS5_ISA virus, NS3NS5_ISA virus, 85 tick-borne encephalitis viruses (TBEV) and 56 other tick-borne flaviviruses (TBFV)

Codon usage bias was evaluated using the effective number of codons (eNC) which gives a value ranging from 20 (only one codon used for each amino-acid) to 61 (random codon usage for each amino-acid). eNC and G+C% values were calculated using the CodonW v1.3 software.

Sequences retrieved from GenBank were detailed in Note A in **S1 Text**.

Virus	Localisation of the re-encoded cassette(s)	Size of the re-encoded cassette(s)	Number of synonymous mutations	eNC	G+C%
**WT_ISA**	-	-	-	53.96	54.3
**NS5 _ISA**	NS5	1,412 nt	273	55.46	53.8
**NS3NS5_ISA**	NS3 - NS5	1,388 nt - 1,412nt	284+273=557	56.41	53.4
**85 TBEV sequences [min ; max]**	-	-	-	[53.33 ; 55.24]	[53.8 ; 55.3]
**56 other TBFV sequences [min ; max]**	-	-	-	[52.35 ; 57.29]	[52.1 ; 55.3]

Table B. Primers and probes used for the real time RT-PCR assays

The TBEV assay detects all TBEVs. The internal control assay system allowed to detect the genome of the MS2 phage (used to spike all samples). The mouse control system targets the housekeeping gene HBS and was used to normalize PCR results obtained with mouse brain samples.

System	Forward/Reverse	Sequence	Nt Position
**TBEV assay**	**Forward**	GCAGAGTGGGCCAGGAACAT	10,235-10,255
	**Reverse**	GTCATGCCGATCCATGCAGGA	10,317-10,337
	**Probe**	FAM-TCGGACAAGAGAAGTTCAAGGACT-TAMRA	10,288-10,311
**Internal control assay**	**Forward**	CTCTGAGAGCGGCTCTATTGGT	-
	**Reverse**	GTTCCCTACAACGAGCCTAAATTC	-
	**Probe**	FAM-TCAGACACGCGGTCCGCTATAACGA-TAMRA	-
**Mouse control**	**Forward**	TGTGTTGCACGATCCTGAAAC	-
	**Reverse**	CTCCTTCCAGGTGCCTCAGAA	-
	**Probe**	FAM-TTCGCTGCATTGCTGAAAGGG-TAMRA	-

Note A.

List of 85 TBEV sequences retrieved from GenBank
GenBank accession numbers: L40361, JQ693478, AY217093, AY182009, GU183379, GU183381, GU183383, GU183384, JQ825146, JQ825161, JQ825144, JQ825158, JQ825159, JQ825164, JQ825154, JQ825156, DQ989336, JX498939, JQ825162, JQ825151, JN229223, JN003205, JF819648, EF469662, EF469661, JF316707, JF316708, JQ825155, JQ650522, JQ650523, EU816451, AF527415, DQ486861, U39292, HM120875, JN003206, AM600965, FJ402885, FJ906622, GU183382, JQ825152, JQ825160, AY169390, EU816452, EU816455, FJ997899, JQ825145, JQ825163, JQ825153, FJ402886, HQ901366, HQ901367, EU816450, HQ201303, JQ825148, JN003207, JQ825147, JQ825150, JX498940, AB062064, DQ153877, AF069066, FJ968751, EU816454, JN003208, JN003209, FJ572210, DQ401140, GQ228395, JQ825157, GQ266392, GU183380, HM535610, HM535611, HM859894, HM859895, JQ825149, EU816453, AB753012, AB062063, GU121642, JX534167, U27491, U27495, DQ862460

List of 56 TBFV sequences retrieved from GenBank
GenBank accession numbers: AF331718, JN860200, JF416949, JF416950, JF416951, JF416952, JF416953, JF416954, JF416955, JF416956, JF416961, JF416962, JF416963, JF416964, JF416965, JF416966, JF416967, JF416957, AF311056, DQ235145, DQ235153, DQ235146, DQ235147, AY863002, DQ462443, AY323490, EU480689, JF416959, JF416960, HM055369,JF416958, AF253419, AF253420, EU790644, Y07863, AY323489, AY193805, AY438626, AB507800, HQ231414, HM440561, HQ231415, HM440562, HM440558, HM440560, HM440559, HM440563, EU670438, EU770575, L06436, DQ235149, DQ235150, DQ235152, DQ235151, DQ235148, EU543649

Note B. Re-encoded sequences

NS3 cassette (corresponding to positions 4,946 – 6,334 of the TBEV genome)

GAGCTGCTTCTGGACACCGGGGGAAGGATTGGGGCGGTGCCAATAGACCTGGCAAAGGGGACTTCTGGCTCCCCTATCCT
GAACAGTCAGGGTGTAGTAGTAGGATTATATGGGAACGGTCTAAAAACAAATGAAACTTATGTTTCATCCATTGCCCAGG
GTGAAGCGGAAAAGTCTCGACCCAATTTGCCGCCTGCAGTAATAGGCACGGGTTGGACAGCCAAGGGCCAGATAACCGT
ACTCGATATGCACCCCGGATCTGGCAAGACACATAGAGTGCTACCGGAACTTATTCGCCAATGTATTGATAGACGCCTTAG
AACACTCGTTCTGGCTCCTACACGTGTGGTGTTGAAGGAGATGGAACGTGCTCTTAGCGGAAAAAGAGTGAGGTTTCATA
GTCCTGCAGTAGGGGATCAACAGGTCGGCGGAGCTATAGTGGACGTCATGTGCCATGCCACTTATGTTAATAGACGCCTA
TTGCCGCAAGGCAGGCAGAACTGGGAAGTGGCCATCATGGACGAAGCCCATTGGACGGACCCACACAGTATAGCTGCTC
GGGGGCACCTATATACTTTGGCAAAGGAGAATAAGTGCGCTCTAGTCTTAATGACGGCTACGCCTCCTGGAAAATCAGAA
CCTTTCCCAGAAAGTAACGGTGCAATATCCAGCGAAGAGAAGCAAATACCAGACGGCGAGTGGCGTGATGGCTTCGACTG
GATAACAGAGTATGAGGGTCGCACAGCATGGTTTGTTCCCTCGATAGCCAAGGGTGGTATCATAGCCCGCACATTGAGAC
AGAAAGGGAAAAGCGTAATTTGCCTCAACAGTAAAACTTTTGAGAAGGATTACTCTAGAGTAAGAGACGAGAAGCCTGAT
TTCGTGGTAACTACAGATATTTCAGAAATGGGTGCTAATTTGGACGTTTCACGTGTTATTGACGGGCGAACAAATATAAAA
CCGGAAGAAGTTGATGGGCGAGTCGAGCTAACAGGTACTAGGCGTGTAACCACGGCATCGGCGGCACAACGCCGTGGTA
GAGTCGGGAGACAGGAGGGCAGGACAGATGAGTATATTTATTCAGGCCAGTGCGATGACGACGATGGTGGGCTTGTTCA
ATGGAAGGAAGCGCAGATCCTGCTCGATAATATCACTACACTGCGGGGACCCGTAGCCACTTTTTATGGTCCCGAACAGG
ATAAAATGCCTGAAGTAGCCGGTCATTTTCGCCTGACAGAGGAGAAAAGGAAGCATTTCCGACACCTACTAACCCATTGTG
ATTTTACGCCGTGGCTAGCTTGGCACGTAGCTGCAAACGTCTCCTCTGTCACATCCCGGAACTGGACGTGGGAGGGACCA
GAGGAGAACACCGTTGATGAAGCTAACGG

NS5 cassette (corresponding to positions 8,619 – 10,019 of the TBEV genome)

GCGAGTTTAATTAACGGAGTTGTTAAGCTCTTATCATGGCCTTGGAACGCGCGGGAGGACGTTGTCCGAATGGCAATGAC
CGATACTACCGCCTTTGGGCAGCAGCGAGTATTCAAAGAGAAGGTAGATACCAAGGCCCAGGAGCCCCAGCCAGGGACG
AAGGTGATCATGAGGGCCGTCAATGACTGGATTCTTGAGCGACTTGCCCGAAAGAGTAAGCCTCGGATGTGTAGTAGGG
AGGAATTCATAGCGAAGGTGAAGAGTAACGCGGCTCTTGGGGCCTGGAGCGATGAGCAAAATAGATGGTCATCCGCTAA
AGAGGCCGTCGAGGACCCCGCATTTTGGCAACTGGTGGACGAGGAAAGGGAAAGACATCTGGCTGGAAGGTGCGCACAT
TGTGTCTATAACATGATGGGGAAAAGGGAGAAAAAGCTTGGAGAGTTTGGTGTTGCTAAGGGGAGTCGGGCCATTTGGT
ACATGTGGCTGGGCAGCCGCTTCCTTGAGTTCGAAGCACTTGGATTTCTAAACGAGGATCACTGGGCTTCCAGGGGGAGC
TCTGGATCAGGAGTTGAAGGTATCTCCCTAAATTATTTAGGATGGTACCTAAAGGGTTTGAGCACTCTTGAGGGCGGACTC
TTTTACGCAGATGATACAGCTGGCTGGGACACTAAAGTCACAAATGCTGACCTGGAGGATGAAGAACAGCTCCTGCGTTA
CATGGAAGGGGAGCATAAGCAGCTTGCGGCCACCATTATGCAGAAGGCCTATCATGCTAAGGTTGTCAAGGTGGCACGG
CCCTCCCGAGACGGTGGTTGTATAATGGATGTGATTACTAGAAGAGACCAAAGAGGCTCTGGCCAAGTAGTGACTTATGC
CCTAAACACCCTCACCAATATTAAAGTACAACTGATACGAATGATGGAAGGCGAGGGTGTCATCGAAGCAACGGACGCCC
ATAACCCAAGACTGTTTCGAGTGGAACGATGGCTCAGGGATCACGGGGAGGAACGTCTTGGGAGAATGTTAGTTTCCGG
AGATGACTGTGTAGTCAGACCTGTCGATGACAGGTTCAGTAGAGCGCTATATTTTCTGAACGATATGGCCAAAACAAGAA
AGGATGTAGGCGAGTGGGAACACTCGGTGGGTTTCTCGAATTGGGAGGAGGTTCCTTTTTGCAGTCATCATTTTCACGAAT
TAGTGATGAAAGATGGGCGCGCCTTAATAGTGCCTTGCCGAGACCAAGATGAATTGGTGGGAAGGGCCCGCGTCTCCCCT
GGGTGCGGCTGGTCAGTTCGTGAGACAGCCTGTTTGTCAAAGGCATATGGCCAAATGTGGCTTTTATCCTATTTTCATCGG
CGCGATCTCCGAACGTTAGGTTTCGCTATCTGTTCGGCGGTCCCC
